# Osthole inhibits triple negative breast cancer cells by suppressing STAT3

**DOI:** 10.1186/s13046-018-0992-z

**Published:** 2018-12-22

**Authors:** Xuanxuan Dai, Changtian Yin, Yi Zhang, Guilong Guo, Chengguang Zhao, Ouchen Wang, Youqun Xiang, Xiaohua Zhang, Guang Liang

**Affiliations:** 1 Chemical Biology Research Center, School of Pharmaceutical Sciences, Wenzhou Medical University, Wenzhou, 325035 Zhejiang China; 2 Department of Surgical Oncology, the First Affiliated Hospital of Wenzhou Medical University, Wenzhou, 325035 Zhejiang China; 3 Department of dermatology, the First Affiliated Hospital of Wenzhou Medical University, Wenzhou, 325035 Zhejiang China

**Keywords:** Osthole, STAT3, Triple-negative breast cancer, Cell apoptosis, Xenografts

## Abstract

**Background:**

Triple-negative breast cancer (TNBC) is an aggressive subgroup of human breast cancer. Patients with TNBC have poor clinical outcome as they are non-responsive to current targeted therapies. There is an urgent need to identify new therapeutic targets and develop more effective treatment options for TNBC patients. Osthole, a natural product from *C. monnieri*, has been shown to inhibit certain cancer cells. However, the mechanisms of action as well as its effect on TNBC cells are not currently known.

**Methods:**

We investigated the effect of osthole in cultured TNBC cells as well as in a xenograft model of TNBC growth. We also used a high-throughput proteomics platform to identify the direct binding protein of osthole.

**Results:**

We found that osthole inhibited the growth of a panel of TNBC cells and induced apoptosis in both cultured cells and TNBC xenografts. We used a high-throughput proteomics platform and identified signal transducer and activator of transcription 3 (STAT3) as a potential binding protein of osthole. We further show that osthole suppressed STAT3 in TNBC cells to inhibit growth and induce apoptosis. Overexpressing STAT3 in TNBC reduced the effectiveness of osthole treatment.

**Conclusions:**

These results provide support for osthole as a potential new therapeutic agent for the management of TNBC. Moreover, our results indicate that STAT3 may be targeted for the development of novel anti-TNBC drugs.

**Electronic supplementary material:**

The online version of this article (10.1186/s13046-018-0992-z) contains supplementary material, which is available to authorized users.

## Background

Triple-negative breast cancer (TNBC) is a unique subset of human breast cancer that is characterized by negative estrogen receptor (ER), progesterone receptor (PR) and human epidermal growth factor receptor 2 (HER2) status. TNBC accounts for approximately 15–20% of all breast cancer diagnosis [1]. TNBC is far more aggressive and shows higher rates of relapse compared to other types of breast cancer [2]. Tumor heterogeneity and lack of effective molecular targets contributes to the poor prognosis [3, 4]. Chemotherapy is currently the mainstay of treatment for patients with TNBC. Although chemotherapy is effective in a subgroup of these cancers, it fails in the majority of patients [5, 6]. Hence, there is an urgent clinical need to discover new molecular targets and develop drugs with minimal toxicity to treat patients with TNBC.

Osthole (7-methoxy-8-isopentenoxycoumarin) is a coumarin-derivative extract of *C. monnieri* that has been shown to inhibit many pathological disorders. These include conditions such as allergies and inflammation [7], diabetes [8], as well as liver injury [9]. In addition, osthole has been reported to beneficial inhibitory effects in multiple types of cancer, including hepatic carcinomas [10], leukemia [11], gastric cancer [12], and lung cancer [13]. Specifically in breast cancer cells, osthole inhibits the growth of breast cancer cells, at least in culture [14, 15]. Taken together, the numerous studies conducted to date suggest that osthol possesses the potential to act in an inhibitory role in the progression of malignancies. However, the mechanisms of function and overall cellular effect of osthol toward particular cancers may not be the same. The mechanisms of action as well as its effect on TNBC cells are not currently known.

Here, we have investigated the effect of osthole in cultured TNBC cells as well as in a xenograft model of TNBC growth. We show that osthole inhibits the growth of TNBC cells and induces apoptosis. Using a high-throughput proteomis platform, we report for the first time, that osthole induces apoptosis in TNBC cells through the inactivation of signal transducer and activator of transcription-3 (STAT3) signaling pathway. In addition, osthole inhibited TNBC cell proliferation in mice implanted with TNBC cells. Our findings show that osthole is a therapeutic candidate in the treatment of patients with TNBC. We have also discovered a novel mechanism of the anti-cancer activities of osthole.

## Methods

### Reagents

Osthole (purity > 99%) and biotin were purchased from the Aladdin Chemicals (China) and was dissolved in DMSO. Biotinylated-osthole (purity > 97.8%) was designed and synthesized by Bocong Biotech (Guangzhou, China). Antibodies against cleaved-PARP (sc-56,196), Bax (sc-493), Bcl-2 (sc-492), Bcl-xl (sc-8392), MDM-2 (sc-965), CyclinB1 (sc-245), CDC2 (sc-54), Ki67 (sc-7846), GAPDH (sc-32,233), horseradish peroxidase (HRP)-conjugated goat anti-mouse IgG, HRP-conjugated donkey anti-rabbit IgG, and PE-conjugated secondary antibodies were purchased from Santa Cruz Biotechnology (Santa Cruz, CA). Antibodies against Phospho-STAT3 (Tyr705, Clone D3A7, 9145), STAT3 (12640S), and cleaved-caspase3 (9661S) were purchased from Cell Signaling Technology (Danvers, MA, USA). Fluorescein isothiocyanate (FITC) Annexin V Apoptosis Detection Kit I and Propidium Iodide (PI) were purchased from BD Pharmingen (Franklin Lakes, NJ).

### Cells culture

Human breast cancer cell lines (MDA-MB-231, BT-549, MDA-MB-468, and MCF-7 were purchased from the Institute of Biochemistry and Cell Biology, Chinese Academy of Sciences (Shanghai, China). MDA-MB-231 and MCF-7 cells were cultured in DMEM medium (Gibco, Eggenstein, Germany), BT-549 cells were cultured in RPMI-1640 medium (Gibco), and MDA-MB-468 were grown in L15 medium (Gibco). Media in all cases was supplemented with 10% heat-inactivated fetal bovine serum (Hyclone, Logan, UT), 100 units/ mL penicillin, and 100 μg/mL streptomycin.

### Cell viability assay

Human breast cancer cells were seeded in 96-well tissue culture plates at a density of 8000 per well, and allowed to attach overnight in complete growth media. Osthole were dissolved in DMSO and then diluted in medium to the desired final concentration (6.25, 12.5, 25, 50,100, 200, 400, and 800 μM). The following day, cells were treated with osthole at increasing concentrations for 24 h, 48 h, or 72 h, respectively. Cell viability was then measured through MTT assay.

### Apoptosis and cell cycle analysis

Cells were plated in 60-mm dishes and allowed to attach overnight. Cells were then treated with osthole at 100, 150, or 200 μM. Following treatments, cells were fixed then labeled with FITC-conjugated Annexin V/PI (for apoptosis detection) or PI staining (for cell cycle detection). Analyses were performed using FACSCalibur flow cytometer. Data for apoptosis and cell cycle distribution was analyzed using FlowJo7.6 software.

To assess morphological changes associated with apoptosis, we stained cells with Hoechst 33258 (Beyotime Biotechnology, China). Cells were challenged with osthole at 100, 150, or 200 μM for 24 or 48 h. Cells were then fixed with 4% formaldehyde solution and stained with Hoechst 33258. Images were captured using a fluorescence microscope (Nikon, Japan). Five microscopic fields were randomly selected from each treatment group.

### Western blot analysis

TNBC cells and tumor tissues were lysed and protein concentrations were measured by the Bradford assay (Bio-Rad, Hercules, CA). Proteins were separated by sodium dodecyl sulfate-polyacrylamide gel electrophoresis and electro-transferred to poly-vinylidene difluoride transfer membranes. Membranes were blocked for 1.5 h at room temperature using fresh 5% nonfat milk in TBST. Primary antibody incubations were carried out overnight at 4 °C. HRP-conjugated secondary antibodies were added for 1 h, and the bands were visualized by using ECL substrate (Bio-Rad). Densitometric measurements were performed using Image J (National Institute of Health, MD).

The cytoplasmic and nuclear extracts were prepared by using KeyGen biotech Nuclear and Cytoplasmic Extraction Reagent kit (Nanjing, China). Cells were washed with ice cold PBS, collected and mixed with Buffer A and Buffer B. The mixture was centrifuged for 10 min at 3000 xg at 4 °C. Supernatant was collected as cytosolic extract. Buffer C was added to the pellets for 1 h. Samples were then centrifuged at 14,000×g for 30 min to extract nuclear proteins. Protein concentrations by measured by the Bradford assay.

### Proteome microarray assay and data analysis

Arrayit HuProt™ v2.0 19 K Human Proteome Microarrays (CDI Laboratories, Baltimore, MD) were blocked with 3% BSA for 1 h at room temperature. Biotinylated–osthole was diluted to 10 μM in blocking buffer and incubated on the proteome microarray for 1 h at room temperature. The arrays were washed with PBST and incubated with Cy3-Streptavidin at 1:1000 dilution (Sigma-Aldrich) for 1 h at room temperature. Finally, the microarray was spun dry and scanned with a GenePix 4200A microarray scanner (Molecular Devices, San Jose, CA). Data were analyzed by GenePix Pro 6.0 software. The signal to noise ratio (SNR) was defined as the ratio of median foreground value minus median background value. A cutoff was of SNR ≥ 1.1 was set.

### Immunofluorescence staining

Cells were seeded in 35-mm cell culture dishes with glass bottom (NEST, Wuxi, China). After overnight culture in complete media, cells were serum-starved for 24 h. Cells were then treated with osthole for 32 h, followed by stimulation by 50 ng/mL IL-6 for 30 min. Cells were fixed in 4% paraformaldehyde/0.1% Triton-X100 solution. Primary anti-p-STAT3 antibody was added and samples incubated at 4 °C overnight. PE-conjugated goat anti-rabbit secondary antibody (1:200) was then added for 1 h. Cells were counterstained with DAPI. Images were captured using a fluorescence microscopy (Nikon, Japan).

### Cell transfections

Cells were transfected with expression-ready pCMV3 vector encoding STAT3 (HG10034-CF, Sino Biological, Beijing, China). To express STAT3, MDA-MB-231 cells were seeded at 6 × 10^5^ cells per dish into 60 mm plates. DMEM medium without antibiotics was added and cells were allowed to grow to 50–70% confluence. The medium was then changed to freshly prepared Opti-MEM medium. Four μg of STAT3 plasmid or control plasmid was dissolved in 6 μL Lipofectamine 2000 reagent and added onto cells. After 6 h, the medium was replaced with complete media containing 10% FBS. P-STAT3 and STAT3 expression in MDA-MB-231 cells was confirmed by Western blotting analysis after 48 h.

### Breast cancer xenografts

All animal studies were in compliance with the Wenzhou Medical University’s Policy on the Care and Use of Laboratory Animals. Protocols for animal studies were approved by the Wenzhou Medical College Animal Policy and Welfare Committee. Five-week-old athymic BALB/c nu/nu female mice (18-22 g) were purchased from Vital River Laboratories (Beijing, China). Animals were housed at a constant room temperature with a 12 h:12 h light/dark cycle and fed a standard rodent diet and given water ad lib. The mice were divided into three experimental groups with six mice in each group. MDA-MB-231 cells were injected subcutaneously into the right flank at 5 × 10^6^ cells in 100 μL of PBS per mouse. When tumors reached a volume of 50–150 mm^3^, mice were treated with osthole by intraperitoneal injections twice daily (100 or 200 mg/kg/d). Osthole was dissolved in 6% castor oil. Tumor volumes were determined by measuring length (l) and width (w) and calculating volume (V = 0.5 × l × w^2^) at the indicated time points. At the end of the study, mice were sacrificed and the tumors were removed. Heart, kidney, and liver tissues were also harvested to assess any toxicity associated with osthole treatment.

### Tissue staining

All harvested tissues were fixed in 10% formaldehyde and embedded in formalin. Tissues were sectioned at 5-μm thickness. Heart, liver and kidney tissues were stained with hematoxylin and eosin (H&E). Tumor tissue sections were deparaffinized, rehydrated and incubated with primarily Ki-67, Bcl-2, Cleaved-caspase 3, MDM2 and CDC2 antibodies. HRP-conjugated secondary antibodies and diaminobenzidine (DAB) were used for detection.

### Statistical analysis

Data shown as mean ± SEM with *n* ≥ 3 independent samples. Statistical analysis was performed with GraphPad Prism 5.0 software (San Diego, CA, USA). One-way ANOVA followed by Dunnett’s post hoc test was used for comparing more than two groups of data, and one-way ANOVA, non-parametric Kruskal–Wallis test, followed by Dunn’s post hoc test was used when comparing multiple independent groups. All other results were analyzed using t-test. Values of *P* < 0.05 were considered statistically significant. Post hoc tests were run only if F achieved *P* < 0.05 and there was no significant variance in homogeneity.

## Results

### Osthole effectively suppresses cell viability by inducing apoptosis in human TNBC cells

**Fig. 1:**
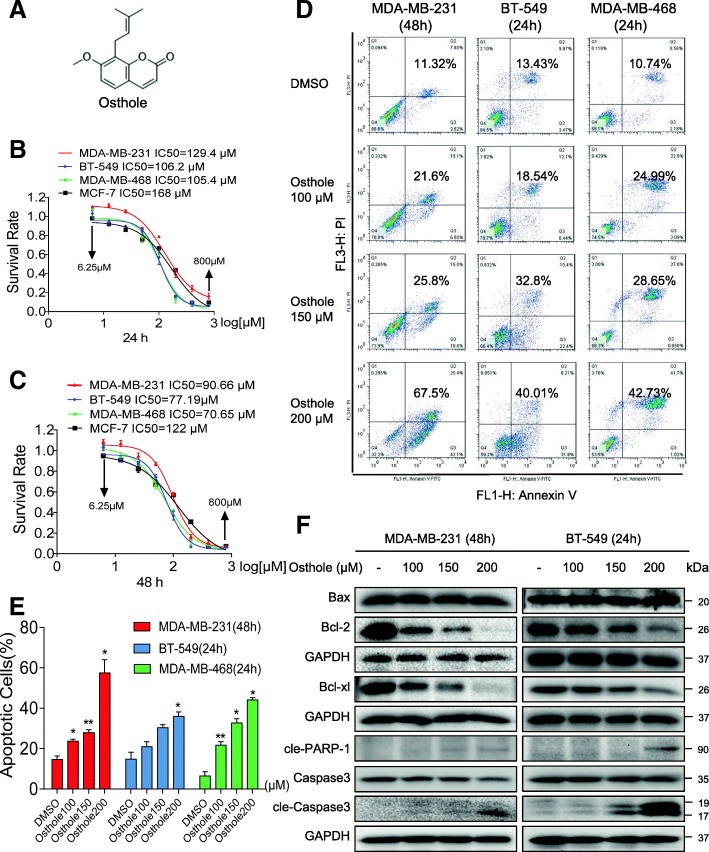
Osthole inhibits cell growth and induces apoptosis in TNBC cells. **a** Chemical structure of osthole. **b, c** The effects of osthole on the viability of human TNBC cells. MDA-MB-231, BT-549, MDA-MB-468, and MCF-7 cells were challenged with increasing concentrations of osthole. Cell viability was determined by MTT assay and the IC_50_ values at 24 h (**b**) and 48 h (**c**) were calculated. **d** Induction of apoptosis in TNBC cells as determined by annexin V/PI staining following exposure to osthole. **e** Quantification of annexin V/PI staining showing the percentage of apoptotic cells following osthole challenge [*P < 0.05, **P < 0.01 and ***P < 0.001 compared to DMSO control]. **f** MDA-MB-231 and BT-549 cells were challenged with osthole for 48 h or 24 h, respectively. Cell lysates were then subjected to assessment of apoptosis-related proteins by western blot. GAPDH was used as internal control. Data representative of 3 independent experiments

Osthole has been reported to exhibit inhibitory activity in a number of human cancers. To investigate whether osthole exhibits similar inhibitory role in breast cancer cells, we first assessed the viability of human breast cancer cells following exposure to osthole. TNBC cells (MDA-MB-231, BT-549, and MDA-MB-468 cells) and non-TNBC cells (MCF-7) were challenged with increasing concentrations of osthole, and the numbers of viable cells were measured by the MTT assay. Our results show that exposure of these breast cancer cells to osthole reduced viability of cells in a dose-dependent manner, at 24 h, 48 h, and 72 h (Fig. 1B, C, and Additional file 1: Figure S1A). The IC_50_ values were found to be 129.4, 106.2, 105.4, and 168 μM at 24 h for MDA-MB-231, BT-549, MDA-MB-468, and MCF-7, respectively. Longer-term exposure for 48 h or 72 h appeared to be more effective as can be seen by reduced IC50 values. In addition, osthole was less cytotoxic and had higher IC_50_ values in MCF-7 cells than in TNBC cells.

Reduced viability in TNBC cells following osthole exposure prompted us to determine whether osthole was inducing apoptosis. Based on our results showing that BT-549 cells are more sensitive to osthole compared to MDA-MB-231, we selected 48 h timepoint for MDA-MB-231 and 24 h for BT-549 to assess apoptosis. Osthole exposure at these timepoints showed nuclear morphological changes illustrative of apoptotic cell death, including nuclear condensation and fragmentation (Additional file 1: Figure S1B). Staining of cells with annexin V/PI showed induction of cellular apoptosis in all three TNBC lines after osthole exposure (Fig. 1d and e). We confirmed these findings by detecting apoptosis-related proteins in TNBC cells. Our results show that osthole decreased Bcl-2 and Bcl-xl (Fig. 1f). In addition, Bax was unaltered in MDA-MB-231 and BT-549. Decreased Bcl-2: Bax ratio signified induction of apoptosis in cells. Figure 1f also showed that osthole treatment increased the levels of cleaved PARP1 and cleaved caspase 3 in TNBC cells.

### Osthole causes cell cycle arrest in TNBC cells

**Fig. 2:**
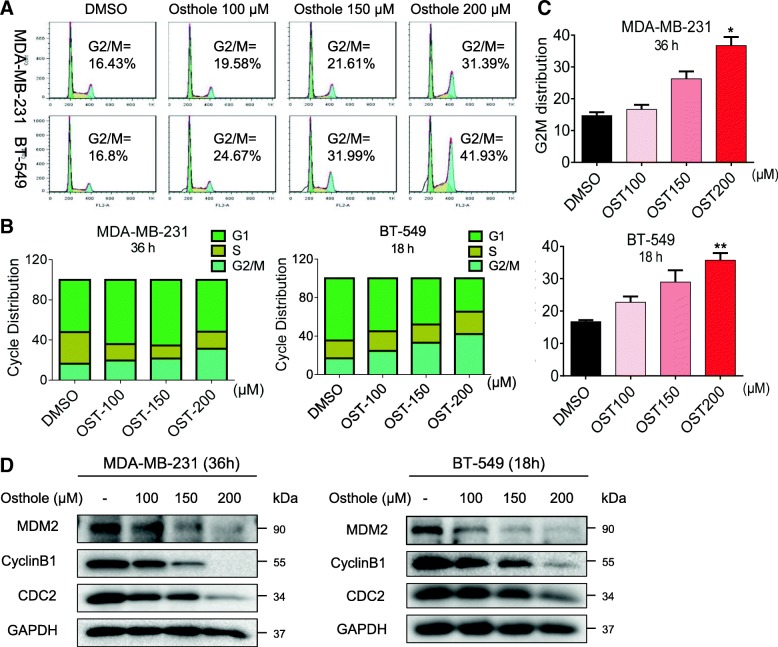
Osthole induces cell cycle arrest in TNBC cells. **a** Induction of cycle arrest in TNBC cells was determined by PI staining and flow cytometry. Cells were exposed to osthole for 36 h (MDA-MB-231) or 18 h (BT-549). **b** The distribution of cell cycle phase in cells challenged with osthole. **c** Representative histograms from flow cytometric analysis in the three TNBC cells treated with osthole [*P < 0.05 and **P < 0.01 compared to DMSO control]. **d** MDA-MB-231 and BT-549 cells were exposed to 200 μM osthole for 36 h and 18 h, respectively. Levels of G2/M cell cycle-related proteins MDM-2, Cyclin B1 and CDC2 were determined by western blot. GAPDH was used as internal control. Data representative of 3 independent experiments

We next determined the effect of osthole on growth of TNBC cells. We treated the cells with increasing concentrations of osthole and assessed cell cycle phase distribution. Osthole induces G2/M cell cycle arrest in MDA-MB-231 as well as BT-549 (Fig. 2a-c). We observed accumulation of cells in the G2/M phase upon 150 μM osthole exposure in MDA-MB-231 and BT-549 cells. Greatest accumulation in G2/M phase was noted with 200 μM osthole. G2/M is an important DNA damage checkpoint and involves a number of regulatory proteins. Key among them are MDM2, Cyclin B1, and CDC2. We assessed the level of these proteins in TNBC cells exposed to osthole and show that osthole causes a decrease in the levels of MDM-2, Cyclin B1, and CDC2 (Fig. 2d). Although the Tyr^15^ phosphorylation of CDC2 is also important for the G2/M phase, we found that osthole induced the remarkable decrease of cdc2 expression. Together, these data reveal that osthole-induced reduced viability of TNBC involves G2/M phase arrest and apoptotic cell death.

### Proteomic identification of osthole binding proteins

**Fig. 3:**
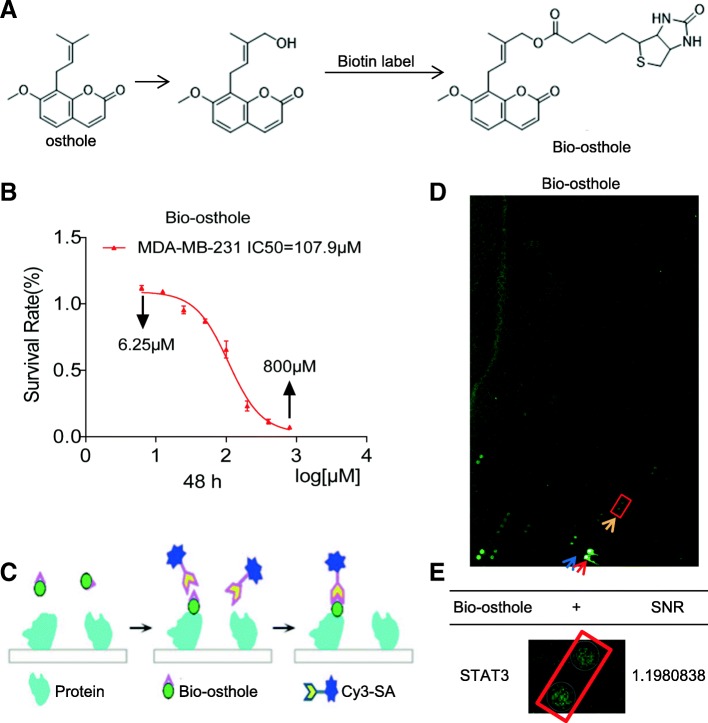
Identification of osthole binding proteins. **a** Chemical structure of osthole intermediate and Biotin-labeled osthole. **b** Viability of MDA-MB-231 cells exposed to biotin-labeled osthole as determined by MTT assay. **c** A schematic illustrating the steps for identifying Bio-osthole binding to proteins fabricated on a microarray. **d** Representative image of an experimental microarray [Blue = negative control, red = positive control, yellow = positive spot]. **e** Magnified image of Bio-osthole binding to recombinant STAT3 protein spot on the microarray Signal to noise ratio (SNR) value is shown

To identify the mechanism and targets underlying the inhibitory osthole activity, we screened for potential osthole binding proteins. To do this, we first generated biotinylated osthole (Bio-osthole). Osthole does not contain modifiable sites such as hydroxyl (-OH) and carboxyl (-COOH), thus we added hydroxyl at the 7- or 8-position isoprenyl ends of osthole to link biotin and obtain biotinylated osthole (Fig. 3a). To ensure that the generated Bio-osthole retained its inhibitory activity in TNBC, we assessed cell viability and compared the results to osthole. We found that the IC_50_ values of Bio-osthole in MDA-MB-231 cells was 107.9 μM, similar closer to the IC_50_ (90.66 μM) of osthole (Fig. 3b). Therefore, Bio-osthole was selected for subsequent protein microarray study. We then used a human proteomic microarray containing 19,394 affinity purified N-terminal GST tagged proteins and covering approximately 75% of the human proteome with Bio-osthole. Briefly, microarrays were probed with Bio-osthole and binding was detected with a Cy3-conjugated streptavidin (Cy3-SA) (Fig. 3c). We then calculated the signal to noise ratio (SNR) for each spot, which was defined as the ratio of median foreground minus median background. We found 199 osthole binding proteins (SNR maximum 1.6178193) (Fig. 3d). Of these proteins, we found STAT3 (SNR 1.1980838) (Fig. 3e) to be an osthole binding protein. Recent studies have demonstrated that STAT3 is abnormally activated in a variety of malignancies, including TNBC. In addition, inhibition of STAT3 can promote apoptosis in human cancers. Taken together, we speculated that STAT3 might be the potential target protein of osthole in generating the inhibitory growth effect in TNBC cells.

### Osthole inhibits constitutive and interleukin-6 (IL-6)-induced STAT3 activity

**Fig. 4:**
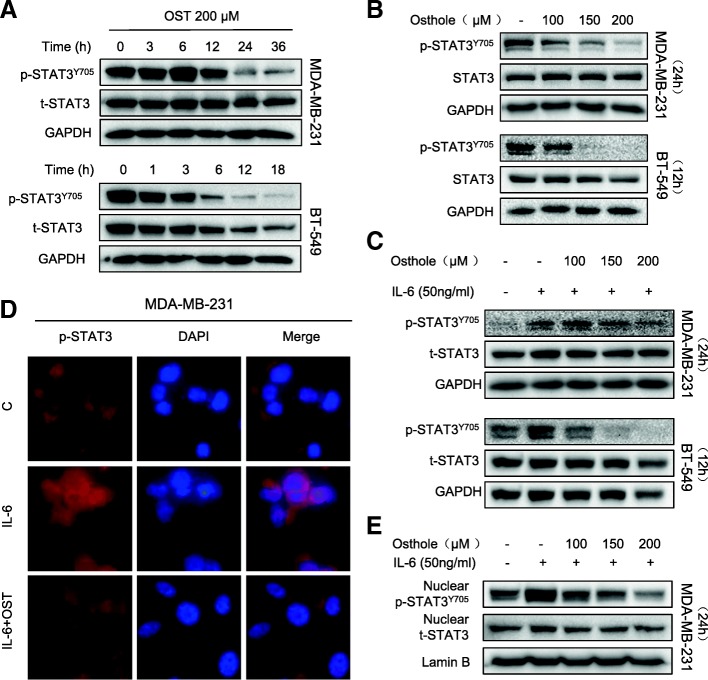
Osthole inhibits STAT3 phosphorylation in TNBC cells. **a** MDA-MB-231 and BT-549 cells were treated with 200 μM osthole for the indicated times, and levels of P-STAT3 were determined by Western blot analysis. GAPDH and STAT3 were used as internal control. **b** MDA-MB-231 and BT-549 cells were exposed to osthole at indicated concentrations for 24 h or 12 h, respectively. P-STAT3 levels were determined by immunoblotting. GAPDH and STAT3 were used as internal control. **c** Cells were pretreated with 200 μM osthole for 24 h (MDA-MB-231) or 12 h (BT-549) and then stimulated with IL-6 (50 ng/mL) for 30 mins. STAT3 phosphorylation was determined by western blot. **d** Immunofluorescence staining of cells showing distribution of P-STAT3 (red) in MDA-MB-231 cells. DAPI was used as counter stain. **e** MDA-MB-231 cells were pretreated with osthole for 24 h before exposure to IL-6 (50 ng/mL) for 30 min. Nuclear extracts were subjected to P-STAT3 and STAT3 immunoblotting. Lamin B was used as loading control

As our analysis of osthole binding proteins revealed STAT3 as a candidate, we assessed the activity of STAT3 in TNBC cells. We found high basal levels of STAT3 phosphorylation (p-STAT3) at tyrosine 705 in in MDA-MB-231 and BT-549 cell lines (Fig. 4a and b). Exposure of cells to osthole reduced the levels of P-STAT3 but not total STAT3 proteins in both a time- and dose-dependent manner (Fig. 4a and b). These findings suggest that osthole may inhibit the activity of STAT3 through direct binding.

Interleukin-6 (IL-6) is known to stimulate STAT3 phosphorylation on tyrosine Tyr705 in many cancer cells. We performed western blotting to determine whether osthole is able to inhibit IL-6-mediated phosphorylation of STAT3. Indeed, osthole inhibits p-STAT3 induced by IL-6 in a dose-dependent manner (Fig. 4c). This inhibition can also be appreciated by immunofluorescence staining of cells with p-STAT3 antibody following IL-6 exposure (Fig. 4d). Furthermore, nuclear extracts prepared from cells show reduced levels of nuclear STAT3 in MDA-MB-231 cells exposed to IL-6 and osthole (Fig. 4e). These results show that osthole effectively prevents the activation and nuclear translocation of STAT3.

### STAT3 overexpression rescued osthole-mediated cytotoxic effects in MDA-MB-231 cells

**Fig. 5:**
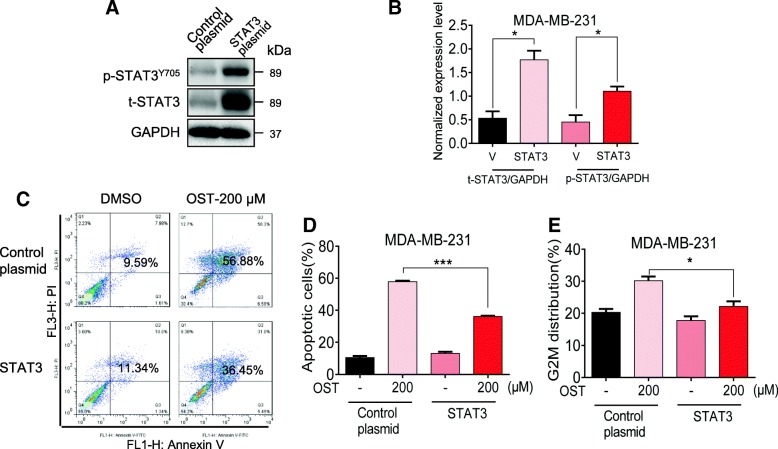
STAT3 overexpression rescued osthole-mediated cytotoxic effects in MDA-MB-231 cells. **a** Representative western blot showing levels of STAT3 and P-STAT3 in MDA-MB-231 cells following transfection with STAT3 plasmid [Control plasmid = control vehicle vector, STAT3 = STAT3 plasmid]. **b** Quantification of STAT3 protein levels from panel A. [*P < 0.05 compared to control plasmid (V)]. **c** STAT3 overexpressing cells and vector control transfected cells were exposed to 200 μM osthole for 48 h, and apoptotic cells were determined by Annexin V/PI staining. **d** Quantification of annexin V/PI staining showing the percentage of apoptotic cells from panel C [***P < 0.001]. (E) STAT3 overexpressing cells and vector control transfected cells were exposed to 200 μM osthole for 36 h, and representative histograms about G2/M cell cycle phase in cells were determined by flow cytometric analysis [*P < 0.05]

We next confirmed the involvement of STAT3 in osthole-induced cytotoxic effects by overexpressing STAT3. Overexpression of STAT3 was utilized to enhance the level of phosphorylated STAT3. We transfected cells with STAT3 expressing plasmid to increase STAT3 and p-STAT3 levels in MDA-MB-231 cells (Fig. 5a and b). Our results show that overexpression of STAT3 reversed osthole-induced apoptosis and cell G2/M cycle arrest in MDA-MB-231 cells (Fig. 5c-e). These findings demonstrate that the inhibitory activity of osthole in TNBC cells is, at least partly, mediated through the inhibition of STAT3.

### Osthole suppressed tumor growth and STAT3 phosphorylation of TNBC cells in vivo

**Fig. 6:**
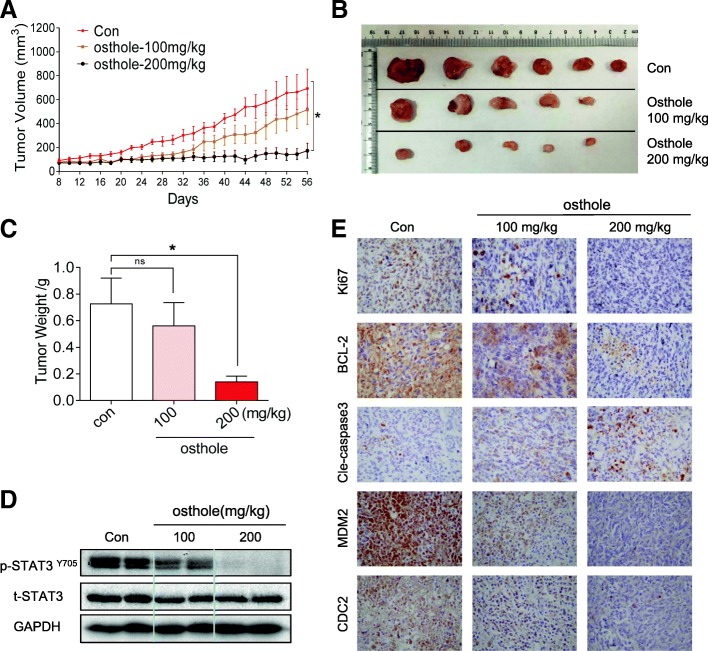
Osthole inhibits MDA-MB-231 xenograft growth in vivo. **a** Tumor volume in vehicle- and osthole-treated mice. MDA-MB-231 cells were injected in the flanks of mice and tumors were allowed to develop for approximately 8 d (50–150 mm^3^). Mice bearing MDA-MB-231 xenografts received osthole at 100 or 200 mg/kg interperitoneally [*P < 0.05]. **b** Images of resected tumor tissues at day 48. Two mice (one in 100 mg/kg osthole group and the other in 200 mg/kg osthole group did not have a visible tumor after 48 d treatment. **c** Tumor weights determined on day 48 [*P < 0.05 compared to vehicle control]. **d** Western blot analysis of P-STAT3 levels in resected tumor specimens. GAPDH was used as loading control. **e** Immunohistochemical staining of tumor sections for cell proliferation marker Ki-67, apoptosis markers Bcl-2 and cleaved caspase-3, and cell cycle markers MDM2, and CDC2. Representative images are shown

Our final objective was to determine whether osthole inhibits the growth of TNBC cells in vivo and whether this inhibition is also mediated through STAT3. We utilized MDA-MB-231 breast cancer cell xenografts in nude mice for these studies. We treated the mice with osthole by intraperitoneal injections at two different doses, 100 or 200 mg/kg. As shown in Fig. 6a-c, treatment with osthole resulted in a significant reduction in both tumor volume and weight compared to the vehicle group. We also noted that the difference of the size of tumors in the control group is relatively big, while the tumor sizes are statistically significant when comparing the control group with osthole-treated group. It is important to note that two mice in the osthole group had no detectable and measurable tumor after the treatment. No significant changes in body weights were noted in any of the experimental groups (Additional file 1: Figure S2A). H&E staining analyses of heart, liver and kidney did not show any toxicity associated with osthole treatment (Additional file 1: Figure S2B).

We then measured the levels of STAT3 in lysates prepared from tumor specimens. Osthole treatment at either 100 or 200 mg/kg reduced the levels of p-STAT3 without altering the levels of total STAT3 (Fig. 6d). Immunohistochemical staining for Ki67 and Bcl2 showed reduced levels of tumor proliferation and growth in osthole-treated mice (Fig. 6e). In addition, levels of cleaved caspase-3 were increased as evident by increased immunoreactivity. We also observed decreased levels of G2/M proteins MDM2 and CDC2 in tumor specimens from mice treated with osthole (Fig. 6e). These results indicate active apoptotic cell death and cell cycle inhibition in tumors treated with osthole. Collectively, our in vivo studies confirm the cytotoxic effects and mechanisms of osthole that we found in our culture studies.

## Discussion

In present study, we found that osthole inhibited the growth of TNBC cells by inducing cell cycle arrest apoptosis. Similarly, osthole treatment of mice bearing MDA-MB-231 TNBC cells showed reduced tumor growth and increased cell apoptosis. We also discovered that osthole mediates these beneficial inhibitory effects in TNBC cells through the suppression of STAT3. Specifically, we demonstrate that osthole binds to and inhibits the phosphorylation of STAT3, thus inhibiting its nuclear translocation. Collectively, these results suggest osthole has potential as a promising candidate for the treatment of TNBC.

A recent in vitro study on two invasive mammary carcinoma cell lines, MDA-MB-231 and 4 T1 cells, showed that osthole inhibited cell proliferation when used in combination with platycodin D [16]. Platycodin D is a triterpene saponin and together with osthole reduced transforming growth factor-β receptor signaling in breast cancer cells. It should be noted that this suppression of signaling was seen in the combination treatment and it is not clear whether osthole along inhibits the pathway. Using a proteomic microarray containing 19,394 proteins, we identified 199 candidate targets to which osthole may bind. One of these candidate binding proteins was STAT3. STAT3 is a member of STAT transcription factors that mediate many aspects of immunity, proliferation, apoptosis and differentiation [17, 18]. This wide range of cellular activities may explain the spectrum of beneficial effects seen with osthole in a variety of disease models. STAT3 is found constitutively phosphorylated in a number of human cancer cell lines and primary tumors [19]. Evidence also suggests that constitutive activation of STAT3 is a point of convergence for malignant transformation at several levels [20], including transformation, proliferation, invasion, and metastasis [21–23]. STAT3 also has been reported to be constitutively active in TNBC [21, 24]. Although there is no difference in the expression levels of STAT3 in ER+, HER2, and TNBC breast cancer subtypes, active and phosphorylated STAT3 has been shown to be restricted to TNBC [25, 26]. Studies have shown that STAT3 signaling is critical for cell survival in TNBC [26–28].

Tumor cells which lack STAT activation are more tolerant to small molecular inhibitors which block STAT3 signal pathway [29–31]. Studies using normal mouse fibroblasts showed that blocking STAT3 signaling causes growth arrest but not apoptosis, suggesting that disruption of STAT3 pathway may not be grossly toxic [32]. However, a large number of malignant transformations with associated constitutive STAT3 activation have been reported [19, 33]. Studies have also shown that the suppression of constitutively active STAT3 leads to growth inhibition and apoptosis in tumor cell lines as well as in xenograft models [24, 34, 35]. Similarly, we found that osthole decreased phosphorylated STAT3 levels in vitro and in vivo. Therefore, STAT3 protein modulation may be an important aspect of the anti-tumor activity of osthole. Moreover, treatment with osthole significantly reduced the levels of phosphorylated STAT3 induced by IL-6 suggesting that osthole selectively inhibits STAT3 phosphorylation. These findings offer the potential for preferential tumor cell killing and make STAT3 an attractive and promising target for therapeutic intervention in human cancer.

## Conclusions

In conclusion, we have identified the anti-tumor activity of osthole against TNBC cells and the potential underlying mechanisms. We found that osthole induced apoptosis and cell cycle arrest in TNBC cells through inhibition of STAT3 phosphorylation and nuclear translocation. This inhibitory activity is partly rescued by STAT3 overexpression. Owing to this inhibitory activity on STAT3 phosphorylation, osthole prevented the proliferatio of TNBC implanted in mice and induced apoptosis. Taken together, our findings show that osthole is a promising candidate for TNBC therapy. Moreover, our results indicate that STAT3 may be targeted for the development of novel anti-TNBC drugs.

## Additional file

Additional file 1: **Figure S1.** showed nuclear morphology of TNBC cells exposed to osthole and **Figure S2.** showed no toxicity in mice administrated with osthole. (PDF 195 kb)

## Abbreviations

Cy3-SA: Cy3-conjugated streptavidin
ER: estrogen receptor
HER2: human epidermal growth factor receptor 2
IL-6: interleukin-6
PR: progesterone receptor
SNR: signal to noise ratio
STAT3: signal transducer and activator of transcription 3
TNBC: triple-negative breast cancer
