# Supplementary material for: Osthole inhibits triple negative breast cancer cells by suppressing STAT3
Source: J Exp Clin Cancer Res. 2018 Dec 22;37:322. doi: 10.1186/s13046-018-0992-z (PMC6303899; doi:10.1186/s13046-018-0992-z)
Supplement: Supplementary file 1 — Figure S1. showed nuclear morphology of TNBC cells exposed to osthole and Figure S2. showed no toxicity in mice administrated with osthole. (PDF 195 kb) [file 13046_2018_992_MOESM1_ESM.pdf]

Supporting Information

Osthole inhibits triple negative breast cancer cells by suppressing STAT3

Xuanxuan Dai[1,2,#], Changtian Yin[1,2,#], Yi Zhang[3], Guilong Guo[2], Chengguang Zhao[1], Ouchen Wang[1], Youqun Xiang[2], Xiaohua Zhang[2,*], Guang Liang[1,*]

[1] Chemical Biology Research Center, School of Pharmaceutical Sciences, Wenzhou Medical University, Wenzhou, Zhejiang 325035, China
[2] Department of Surgical Oncology, the First Affiliated Hospital of Wenzhou Medical University, Wenzhou, Zhejiang 325035, China
[3] Department of dermatology, the First Affiliated Hospital of Wenzhou Medical University, Wenzhou, Zhejiang 325035, China

Running title: Osthole inhibits TNBCs by targeting STAT3

# These authors contribute equally to this work.

***Corresponding authors:**

Guang Liang, Ph.D, Professor
Chemical Biology Research Center, School of Pharmaceutical Sciences, Wenzhou Medical University, Wenzhou 325035, China
Tel: +86-577-86699892; Fax: +86-577-86689982;
E-mail: wzmcliangguang@163.com

Xiaohua Zhang,
Department of surgical Oncology, The First Affiliated Hospital of Wenzhou Medical University, Wenzhou, Zhejiang 325035, China
E-mail: xiaohuazhang2015@sina.com

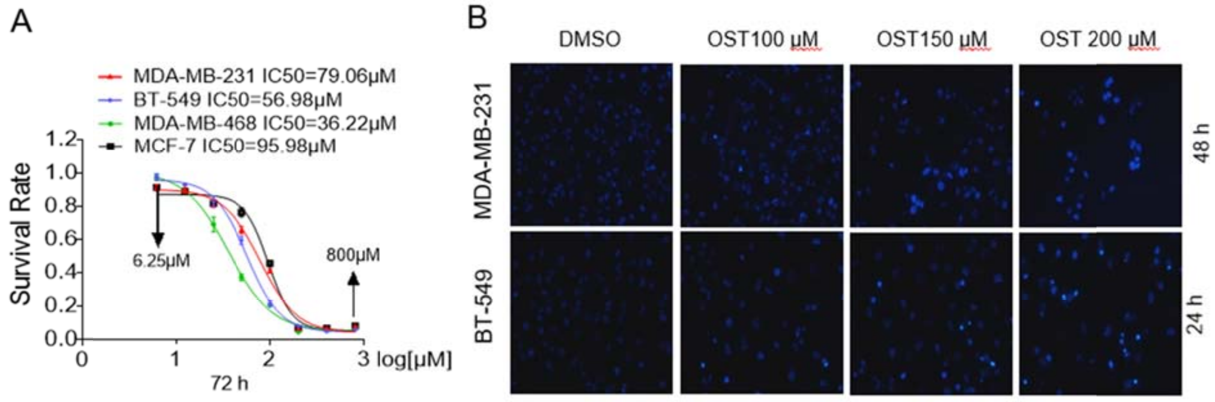

Supplementary Figure S1: Nuclear morphology following osthole exposure in TNBC cells. (A) The effects of osthole on the viability of human TNBC cells. MDA-MB-231, BT-549, MDA-MB-468, and MCF-7 cells were challenged with increasing concentrations of osthole. Cell viability was determined by MTT assay and the IC_{50} values at 72h were calculated. **(B)** MDA-MB-231 and BT-549 cells were treated with increasing concentrations of osthole for 48 h or 24 h, respectively. Cells were then stained with Hoechst 33258 to assess apoptosis-related nuclear morphology.

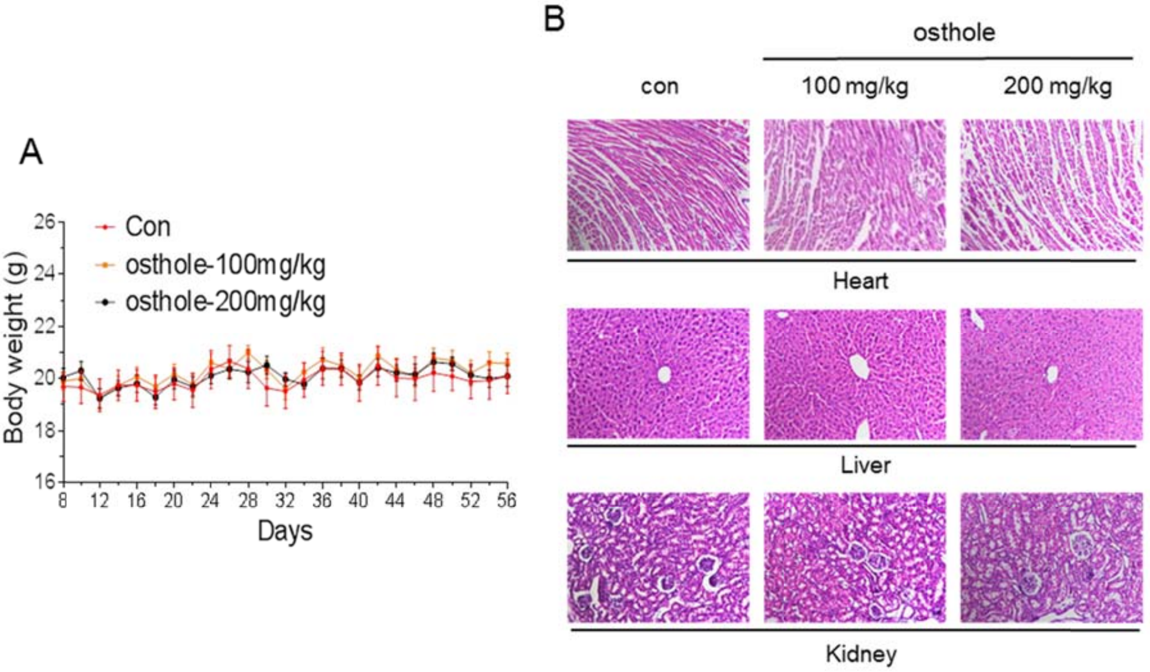

Supplementary Figure S2: Effects of osthole on body weights and tissue structure

(A) Body weights of the mice in vehicle- and osthole-treatment groups. (B) H&E staining of heart, liver, and kidney specimens from mice harboring MDA-MB-231 tumors.
